# Characterization of PBMC secretome: Iron-quercetin preconditioning enhances pro-angiogenic and tissue regeneration factors for potential autologous diabetic wound healing applications

**DOI:** 10.1016/j.bbrep.2025.102338

**Published:** 2025-11-05

**Authors:** Jiraporn Kantapan, Phattarawadee Innuan, Chonticha Sirikul, Nampeung Anukul, Gwenaël Rolin, Nathupakorn Dechsupa

**Affiliations:** a Molecular Imaging and Therapy Research Unit, Department of Radiologic Technology, Faculty of Associated Medical Sciences, Chiang Mai University, Chiang Mai, 50200, Thailand; b Department of Radiologic Technology, Faculty of Associated Medical Sciences, Chiang Mai University, Chiang Mai, 50200, Thailand; c Division of Transfusion Science, Department of Medical Technology, Faculty of Associated Medical Sciences, Chiang Mai University, Chiang Mai, 50200, Thailand; d INSERM CIC-1431, CHU Besançon, Besançon, F-25000, France; e Université Marie et Louis Pasteur, UM RIGHT, Besançon, F-25000, France

**Keywords:** Iron–quercetin complex, Peripheral blood mononuclear cells (PBMCs), Secretome, Diabetes wound healing, Tissue repair, Regenerative medicine, Angiogenesis

## Abstract

Impaired wound healing, particularly in diabetic and chronic wounds, remains a significant global health challenge. While current therapies show promise, their effectiveness is often constrained by high costs, limited accessibility, and inconsistent outcomes across patient populations. This study investigates the potential of the Iron–Quercetin complex (IronQ) to enhance the regenerative secretory profile of peripheral blood mononuclear cells (PBMCs), with the aim of developing a cost-effective, cell-free therapeutic approach. PBMCs isolated from both healthy and diabetic donors were preconditioned with IronQ for 10 days. The resulting secretomes were analyzed for their protein and metabolic profiles, focusing on pro-angiogenic and immunomodulatory factors. In vitro assays were performed to assess the effects of IronQ-preconditioned secretomes on the proliferation, migration, and collagen production of endothelial cells, fibroblasts, and keratinocytes. IronQ preconditioning significantly enriched PBMC secretomes with cytokines such as TGF-α, TNF-α, IL-6, IL-10, HGF, G-CSF, M-CSF, GM-CSF, and GRO-α, along with enhanced glycolytic activity and the production of bioactive metabolites. Furthermore, these secretomes promoted the proliferation and migration of endothelial cells, fibroblasts, and keratinocytes, as well as collagen production by fibroblasts, with consistent effects observed across both healthy and diabetic donors. Collectively, these findings support the potential of IronQ-preconditioned PBMC secretomes as a scalable, autologous, and cost-effective alternative to current cell-based therapies for diabetic wound healing, warranting further in vivo and clinical evaluation.

## Highlights

• IronQ enhances PBMC secretory activity and regenerative signaling.
• IronQ-PBMC secretomes increase angiogenic and immunomodulatory factors.
• IronQ-PBMC secretome promotes cell proliferation, migration, and collagen.
• Effects are consistent in healthy and diabetic donors for autologous use.
• Secretome offers a scalable and cost-effective cell-free wound therapy.

## Graphical abstract

**Figure:**
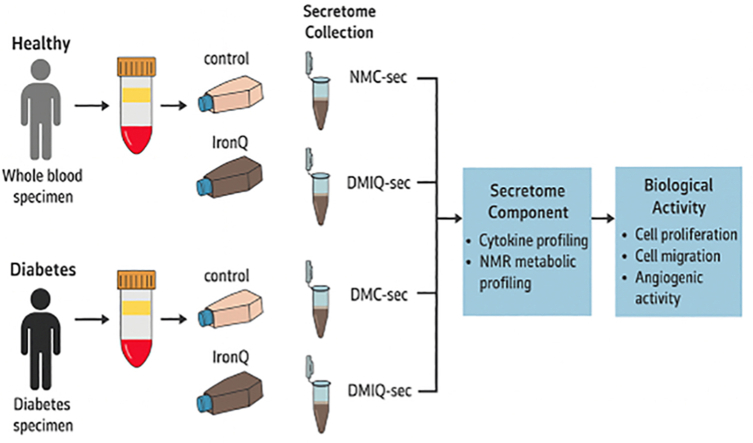

**Table:** Abbreviations

IronQ	Iron-Quercetin complex
PBMCs	Peripheral blood mononuclear cells
MSC	Mesenchymal stem cell
CM	Conditioned media
NMC-sec	Conditioned medium from untreated PBMCs of healthy donors
NMIQ-sec	Conditioned medium from IronQ-treated PBMCs of healthy donors
DMC-sec	Conditioned medium from untreated PBMCs of diabetic donors
DMIQ-sec	Conditioned medium from IronQ-treated PBMCs of diabetic donors
EPCs	Endothelial progenitor cells
DM	Diabetic
HGF	Hepatocyte growth factor
AKI	Acute kidney injury
HDF	Human dermal fibroblasts
HaCaT	Human keratinocyte cell line
HUVECs	Human umbilical vein endothelial cells
ATCC	American Type Culture Collection
DMEM	Dulbecco's Modified Eagle Medium
FBS	Fetal bovine serum
EGM	Endothelial growth medium
EGF	Epidermal growth factor
FGF	Fibroblast growth factor 2
bFGF	Basic fibroblast growth factor
IGF-1	Insulin-like growth factor 1
VEGF	Vascular endothelial growth factor
PE	Phycoerythrin
1H NMR	Proton Nuclear Magnetic Resonance spectroscopy
qRT-PCR	Quantitative real-time polymerase chain reaction
KO	Knockout
Col-1	Collagen type I protein
Col-3	Collagen type III protein
CPMG	Carr-Purcell-Meiboom-Gill
PPI	Protein-protein interaction
GO	Gene ontology
IL-6	Interleukin 6
IL-8	Interleukin 8
IL-10	Interleukin 10
MCP-1	Monocyte chemotactic protein 1
PDGF-AA	Platelet-derived growth factor subunit A
PDGF-AB/BB	Platelet-derived growth factor subunits A and B
VEGF-A	Vascular endothelial growth factor-A
TGF-α	Transforming growth factor alpha
TNF-α	Tumor necrosis factor alpha
GRO-α	Growth-regulated oncogene α
G-CSF	Granulocyte colony-stimulating factor
GM-CSF	Granulocyte-macrophage colony-stimulating factor
M-CSF	Macrophage colony-stimulating factor
COL1A1	Collagen type I alpha 1 chain
COL1A2	Collagen type I alpha 2 chain
COL3A1	Collagen type III alpha 1 chain
GAPDH	Glyceraldehyde 3-phosphate dehydrogenase
M1	Macrophages type 1 with a pro-inflammatory phenotype
M2	Macrophages type 2 with an anti-inflammatory phenotype

## 1 Introduction

Impaired wound healing, especially in conditions like diabetes and chronic wounds, is a major global health issue [1]. Current therapeutic approaches, including stem cells, genetically modified skin cells, and recombinant proteins, have shown promise but are often limited by cost, accessibility, and varying efficacy across patient populations [2,3]. A growing body of evidence indicates that the benefits of many cell therapies arise less from durable engraftment than from paracrine signaling that promotes angiogenesis, restrains excessive inflammation, and supports keratinocyte and fibroblast migration—processes central to tissue repair [[4], [5], [6]]. These insights have focused attention on the therapeutic “secretome,” the complex mixture of cytokines, chemokines, growth factors, lipids, and extracellular vesicles released by cells as a potent tool for regenerative medicine [7,8]. Delivering conditioned medium (CM)—the cell-free form of the secretome—captures the key paracrine activities of cell therapies while avoiding major hurdles of live-cell delivery such as immune incompatibility, tumorigenicity, poor engraftment, and stringent handling [9,10]. As an acellular product, CM can be sterilized, stored (including lyophilized formats), dose-standardized, and batch-tested, enabling scalable, cost-effective manufacturing with consistent quality control [[11], [12], [13]]. These attributes make CM a practical, off-the-shelf option for wound care that mitigates risks associated with cell-based approaches (e.g., GVHD, embolus formation) while retaining therapeutic efficacy. In preclinical and early clinical studies, secretomes derived from mesenchymal stromal cells, endothelial progenitors, and immune cells have accelerated re-epithelialization, enhanced granulation tissue formation, and improved neovascularization, underscoring the translational potential of cell-free biologics [14,15]. Building on this paradigm, conditioned media offer additional advantages relevant to precision care: secretory profiles can be “preconditioned” by biochemical cues or culture conditions to enrich pro-angiogenic or pro-resolving factors; CM can be combined with hydrogels, dressings, or sprayable formulations for controlled release; and its acellular nature may simplify quality control and regulatory pathways compared with living cells [[16], [17], [18]]. Together, these features position conditioned medium as a pragmatic, mechanistically grounded modality to improve healing in difficult-to-treat wounds.

Mesenchymal stem cell (MSC) therapy holds substantial promise, but clinical translation is often constrained by cost, manufacturing complexity, access, and variable efficacy across patient populations [19]. Peripheral blood mononuclear cells (PBMCs) represent a practical alternative. Comprising lymphocytes, monocytes, and circulating progenitors, PBMCs are readily obtained—often from material that would otherwise be discarded—and exert therapeutic effects largely through paracrine signaling [20]. PBMC secretomes have shown protective or reparative activity in models of myocardial infarction [21], autoimmune myocarditis [22], spinal cord injury [23], and cutaneous wound healing [24], reflecting the concerted action of multiple cytokines, chemokines, and growth factors. In the context of diabetic wounds, PBMC-based approaches are minimally invasive and autologous, and their secretomes—enriched in VEGF, HGF, FGF, and TGF-β—promote angiogenesis, stimulate fibroblast function, and accelerate re-epithelialization; anti-inflammatory mediators such as IL-10 further help resolve chronic inflammation that impedes repair [[25], [26], [27], [28], [29]]. However, PBMCs contain a relatively low proportion of regenerative cells, which is particularly problematic in individuals with diabetes or metabolic disorders, where the quantity and functionality of regenerative cells are often diminished [30]. Moreover, the therapeutic efficacy of PBMC secretomes varies with the cell source and culture conditions, affecting their potential benefits [25]. Metabolic dysregulation, oxidative stress, and persistent low-grade inflammation in diabetes blunt PBMC reparative capacity, reducing the effectiveness of autologous products [30,31]. A recent study demonstrated that culturing PBMCs from diabetic patients using a specific QQ culture technique with five cytokines significantly enhances the vasculogenic potential of EPCs derived from PBMCs, showing effectiveness in treating non-healing extremity ulcers. However, this approach is costly due to cytokine requirements and lacks the ability to track transplanted cells [32].

To address these gaps, we explored the paramagnetic iron–quercetin complex (IronQ) as a low-cost, dual-function “theranostic” that both augments PBMC reparative biology and supports MRI-based tracking. IronQ has been validated as a T1-weighted MRI contrast agent with favorable biocompatibility and intracellular uptake [33,34]. Beyond imaging, IronQ promotes differentiation of PBMCs toward pro-angiogenic phenotypes, expanding the limited pool of endothelial progenitor cells (EPCs) relevant to wound repair. Building on this rationale, we preconditioned PBMCs with IronQ to “tune” their secretory output: prior work shows increased release of pro-angiogenic and anti-inflammatory factors and upregulation of angiogenic/immunomodulatory gene programs central to tissue repair [26,35]. This strategy may be particularly advantageous in diabetes, where metabolic dysfunction and oxidative stress compromise progenitor migration and differentiation. Related models further underscore IronQ's therapeutic potential: IronQ-labeled MSCs attenuated neuroinflammation and improved neurological outcomes after intracerebral hemorrhage, and IronQ-preconditioned MSCs exhibited greater therapeutic efficacy than unmodified MSCs in mouse models of cisplatin-induced kidney injury. This enhanced effect is attributed to IronQ-induced secretion of hepatocyte growth factor (HGF) by MSCs, which activates the HGF/c-Met signaling pathway, improving MSCs' ability to treat acute kidney injury (AKI) [[36], [37], [38]].

Here, we compare secretomes from IronQ-preconditioned and non-preconditioned PBMCs from healthy volunteers (NMC-sec vs. NMIQ-sec) and patients with diabetes (DMC-sec vs. DMIQ-sec), identify key soluble factors, and test their capacity to promote angiogenesis and other in-vitro surrogates of wound repair. Our explicit hypothesis is that IronQ preconditioning enhances the pro-regenerative secretome of human PBMCs and improves in-vitro measures of wound repair (e.g., endothelial tube formation, fibroblast migration, and pro-healing cytokine profiles). Ultimately, this work aims to inform an autologous, cell-free therapy for diabetic foot ulcers—an approach that is scalable, cost-effective, and well-suited to patients with chronic wounds and ischemic comorbidities.

## 2 Materials and methods

### 2.1 Human subject

Ten male patients aged 30–70 years, diagnosed with type 2 diabetes and with HbA1C levels below 8.0 g/dL, were recruited from a private healthcare clinic in Chiang Mai province, Thailand. Exclusion criteria included individuals with malignant tumors, those undergoing hemodialysis or peritoneal dialysis, and patients with infectious diseases, hematologic disorders, or severe heart failure. Additionally, ten healthy male volunteers within the same age range were selected as control subjects. To minimize gender-related variability, only male participants were included in the study. The study protocol was approved by the Human Research Ethics Committee of the Faculty of Associated Medical Sciences, Chiang Mai University (protocol no. AMSEC-65EX-023, approved on July 7, 2022). All procedures were conducted in accordance with the guidelines and regulations of the Review Board and adhered to the principles of the Declaration of Helsinki. Informed consent was obtained from all participants prior to their inclusion in the study.

### 2.2 Isolation and cell culture of peripheral blood mononuclear cells (PBMCs)

Whole blood samples (50 mL) were collected from healthy individuals and patients with diabetes mellitus (DM) for the isolation of peripheral blood mononuclear cells (PBMCs) using density gradient centrifugation. Each blood sample was first diluted in phosphate-buffered saline (PBS, pH 7.4) at a 1:1 ratio. The diluted blood was then carefully layered over lymphocyte separation media (Lymphoprep™, Stem Cell Technologies, Vancouver, BC, Canada) and centrifuged, resulting in a distinct mononuclear cell layer. This layer was collected and cultured in Roswell Park Memorial Institute 1640 (RPMI 1640) medium, supplemented with l-glutamine (Caisson Lab, Smithfield, UT, USA), 10 % fetal bovine serum (FBS) (Gibco, Waltham, MA, USA), and 1 % penicillin/streptomycin (Gibco, Waltham, MA, USA). The cultured PBMCs were then incubated at 37 °C in a humidified atmosphere with 5 % CO_2_.

### 2.3 Iron-Quercetin complex (IronQ) preparation

The iron (III)-quercetin complex (IronQ) was synthesized as described in a previous study [33]. IronQ is characterized by a hydrodynamic size of 160.0 ± 2.4 nm in Milli-Q water, with a spherical morphology. It exhibits a negative surface charge, as indicated by a mean zeta potential of −24.53 ± 1.88 mV, which reflects its moderate stability in aqueous suspension. This negative charge helps to extend IronQ's half-life in circulation by creating electrostatic repulsion with plasma proteins. For preparation, IronQ powder was dissolved in distilled water to obtain a stock solution with a final concentration of 2 mg/mL. The solution was then filtered through a 0.2 μm sterile syringe filter to ensure sterility before use.

### 2.4 Treatment of PBMCs with IronQ and preparation of PBMC secretomes

PBMCs were freshly isolated from healthy individuals and diabetic (DM) patients. The cells were seeded in cell culture flasks at a density of 1 × 10^6^ cells/mL in RPMI 1640 medium supplemented with 10 % FBS. IronQ was added at a final concentration of 125 μg/mL, based on previously established optimal conditions for PBMC applications [26]. Cultures were maintained at 37 °C in a humidified atmosphere containing 5 % CO_2_, without subculturing or medium replenishment during the incubation period. Cellular morphology changes following IronQ treatment were monitored at each time point using an inverted microscope.

After a 10-day incubation period, the treated cells were washed twice with PBS to remove residual serum and media components. The cells were subsequently incubated in 10 mL of RPMI 1640 medium without FBS for an additional 24 h to allow for the collection of secreted factors. Conditioned media were harvested after the 24-h incubation by centrifugation at 1500 rpm for 15 min to remove cells and debris. The supernatant was then filtered through a 0.22-μm syringe filter to ensure sterility and the removal of particulate matter. The collected secretomes were categorized as follows:

• NMC-sec: Conditioned medium from untreated PBMCs of healthy donors.
• NMIQ-sec: Conditioned medium from IronQ-treated PBMCs of healthy donors.
• DMC-sec: Conditioned medium from untreated PBMCs of diabetic donors.
• DMIQ-sec: Conditioned medium from IronQ-treated PBMCs of diabetic donors.

**Fig. 1:**
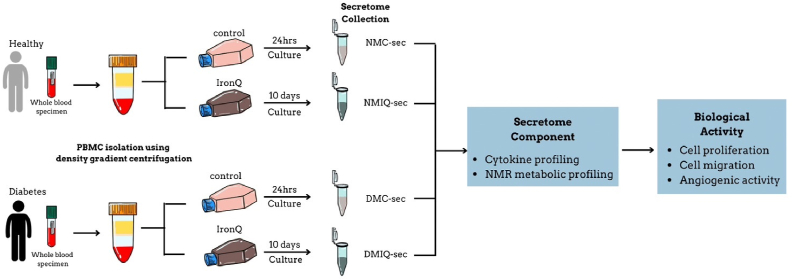
Flow chart demonstrating the workflow of PBMC secretome preparation and its biological activity testing.

To standardize analyses and reduce donor variability, conditioned media (CMs) from 10 different donors were pooled for each experimental group. This workflow ensured reproducibility and accurate representation of PBMC-secreted factors for downstream analyses. A schematic of the study design is shown in Fig. 1.

### 2.5 Cell lines and cell cultures

Human dermal fibroblasts (HDF), human keratinocyte cell line (HaCaT), and human umbilical vein endothelial cells (HUVECs) were obtained from the American Type Culture Collection (ATCC; Manassas, VA, USA). HDF and HaCaT cells were cultured in Dulbecco's Modified Eagle Medium (DMEM; Gibco, Waltham, MA, USA) supplemented with 10 % FBS (Gibco, Waltham, MA, USA), 25 mM l-glutamine (Gibco, Waltham, MA, USA), and 1 % penicillin-streptomycin solution (100 U/mL penicillin, 100 μg/mL streptomycin; Gibco, Waltham, MA, USA). HUVECs were cultured in endothelial growth medium (EGM), a modified DMEM/F-12 medium supplemented with 10 % FBS, 1 % penicillin-streptomycin, and a specialized growth factor cocktail containing 5 ng/mL epidermal growth factor (EGF), 10 ng/mL basic fibroblast growth factor (bFGF), 20 ng/mL insulin-like growth factor 1 (IGF-1), 0.5 ng/mL vascular endothelial growth factor (VEGF), 22.5 μg/mL heparin, and 0.2 μg/mL hydrocortisone. All cells were cultured in an incubator at 37 °C in a humidified atmosphere with 5 % CO_2_.

### 2.6 Characterization of cytokines and growth factors in PBMC secretome

To characterize the cytokines and growth factors in the PBMC secretome, conditioned media from 10 donors were pooled for each experimental group to minimize donor variability and enhance the representativeness of the profiles. The pooled samples were analyzed using the MILLIPLEX® MAP Human Cytokine/Chemokine/Growth Factor Panel A and the MILLIPLEX® MAP Human Angiogenesis/Growth Factor Magnetic Bead Panel (Merck Millipore, USA), which utilize xMAP technology (Luminex, USA) for high-throughput, multiplexed detection of multiple analytes within a single sample. All secretome samples were prepared and analyzed according to the manufacturer's guidelines. Briefly, samples were thawed on ice, gently vortexed, diluted as necessary to ensure analyte concentrations were within the assay's detection range, and added in duplicate. Control (non-conditioned) culture medium was used as background for standard and control wells. Magnetic beads conjugated with analyte-specific antibodies were incubated with the secretome samples, allowing target proteins to bind, followed by washing steps to remove unbound material and the sequential addition of detection antibodies and streptavidin-phycoerythrin (PE) reporter dye for signal generation. Beads were analyzed using a Luminex MAGPIX® instrument with xPONENT (Luminex, USA) software, which measures fluorescence intensities corresponding to the concentration of each analyte. Standard curves were generated for each analyte using reference standards provided with the assay kits, enabling quantification of cytokines and growth factors in pg/mL. Of the 21 analytes assessed, 9 were excluded from analysis due to undetectable expression levels or lack of significant differences between experimental conditions. To further explore the relationships among the identified proteins, a protein–protein interaction (PPI) network was constructed using the STRING database (https://www.string-db.org/). The network was visualized with Cytoscape version 3.8.2 (https://cytoscape.org/).

### 2.7 Characterization of metabolites in PBMC secretome by Nuclear Magnetic Resonance (NMR) spectroscopy

Metabolite content in the PBMC secretome was analyzed using ^1^H NMR spectroscopy. Secretome samples were retrieved from −80 °C storage and thawed at room temperature. A 100 μL aliquot of each sample was combined with 500 μL of deuterium oxide (minimum deuteration degree 99.9 %, CAS number 7789-20-0, Sigma-Aldrich, Merck KGaA, Germany) containing 0.05 wt% sodium trimethylsilyl-[2,2,3,3-d_4_]-propionate (TSP-D4) as an internal reference for calibration and quantification. The mixture was gently mixed and transferred into 5 mm NMR capillary tubes, with 550 μL loaded into each tube. Proton spectra were acquired at 27 °C using a Bruker AVANCE 500 MHz NMR spectrometer (Bruker, Germany) with the Carr-Purcell-Meiboom-Gill (CPMG) pulse sequence (RD–90°–(t–180°–t) n–acquire) for water suppression. A pre-saturation pulse sequence was used, with a 90° pulse and 16 signal averages (NSA) applied. Spectral acquisition, baseline correction, and phase adjustment were performed using TopSpin 4.0.7 software (Bruker, Germany). The spectral region from 0 to 8 ppm was analyzed, with normalization of spectral data to the total integrated area prior to statistical analysis. Metabolite resonances were assigned based on comparison with existing literature and publicly available human metabolite databases.

### 2.8 Cell proliferation assay

HDFs, keratinocytes, and HUVECs were seeded in 96-well plates at an appropriate density and incubated overnight to allow cell attachment. Six replicates were prepared for each experimental group. The cells were then cultured for 72 h with 50 % conditioned medium derived from PBMC secretomes: NMC-sec, NMIQ-sec, DMC-sec, or DMIQ-sec. Normal culture medium served as the control group. After 72 h, 10 μL of CCK-8 solution (Abbkine, Wuhan, China) and 100 μL of fresh culture medium containing 10 % FBS were added to the experimental wells and blank wells. Plates were incubated in the dark at 37 °C for 2 h. Optical density (OD) values were measured at 450 nm using a microplate reader (BioTek^TM^ Eon^TM^ microplate reader, Winooski, VT, USA). Cell proliferation and viability were calculated as the relative OD values of the experimental wells compared to the control wells. This ratio was used to determine the effects of the secretomes on cellular proliferation and viability.

### 2.9 Cell migration by a scratch wound healing assay

The migratory effects of PBMC secretomes on cells were assessed using a scratch wound healing assay. Confluent cell monolayers were established in a cell culture system by seeding cells and maintaining them until full confluency was achieved. A sterile pipette tip was used to create a uniform scratch (wound gap) across the monolayer, simulating a wound area. The wells were then washed with PBS to remove detached cells and debris. Fresh serum-free medium containing 50 % conditioned medium from PBMC secretomes (NMC-sec, NMIQ-sec, DMC-sec, or DMIQ-sec) was added to each well. The plates were incubated at 37 °C in a humidified atmosphere with 5 % CO_2_. Images of the wound gap were captured at 0, 24, and 48 h using a microscope (Nikon, ECLIPSE Ts2, Tokyo, Japan). The migration of cells into the wound area was analyzed by measuring the reduction in the wound gap using ImageJ software 1.52v version (National Institute of Health, Bethesda, MD, USA). The results quantified the extent of wound closure over time, reflecting the migratory capability of cells treated with the different PBMC secretomes.

### 2.10 Cell migration assay using transwell chambers

The migratory effects of PBMC secretomes on cells were evaluated using Millicell® 24-well cell culture insert plates with 8-μm pore Transwell chambers (Merck Millipore, USA). Cells were cultured in serum-free medium for 24 h to induce serum deprivation and then resuspended in serum-free medium. The cell suspension was seeded into the upper chamber of the Transwell insert. The lower chamber was filled with 700 μL of fresh culture medium supplemented with 50 % conditioned medium derived from PBMC secretomes (NMC-sec, NMIQ-sec, DMC-sec, or DMIQ-sec). The plates were incubated at 37 °C in a humidified atmosphere with 5 % CO_2_ for 24 h to allow cell migration through the pores. After incubation, the cells on the upper surface of the membrane were carefully removed using cotton swabs. Migrated cells on the lower surface were fixed with 4 % paraformaldehyde in PBS and stained with crystal violet for 15 min. The number of migrated cells on the lower membrane surface was visualized and quantified using an inverted microscope (Nikon, ECLIPSE Ts2, Tokyo, Japan) by counting cells in representative fields. All experiments were performed in triplicate to ensure reproducibility. The results were reported as the relative number of migrated cells per field.

### 2.11 In vitro tube formation assay

The ability of PBMC secretomes to promote capillary-like structure formation was assessed using a Matrigel basement membrane matrix (Thermo Fisher Scientific, Waltham, MA, USA). Matrigel was used to coat 96-well culture plates according to the manufacturer's instructions and allowed to polymerize. HUVECs were seeded at a density of 5 × 10^4^ cells per well in a mixture of 50 % conditioned medium derived from PBMC secretomes (NMC-sec, NMIQ-sec, DMC-sec, or DMIQ-sec) and 50 % HUVEC medium. EGM medium was used as the control. The cells were incubated at 37 °C in a humidified atmosphere with 5 % CO_2_, and tube formation was monitored at 4, 8, and 12 h using an inverted microscope (Nikon, ECLIPSE Ts2, Tokyo, Japan). The number of capillary-like structures formed was quantified by counting tubes in five randomly selected fields per well. The results were expressed as the mean ± standard deviation (SD) of three independent experiments.

### 2.12 Quantitative real-time polymerase chain reaction (qRT-PCR)

Total RNA was extracted from human dermal fibroblast cells treated with PBMC secretomes using the NucleoSpin® RNA Kit (Macherey–Nagel, Düren, Germany) according to the manufacturer's instructions. The isolated RNA was then reverse transcribed into complementary DNA (cDNA) using the ReverTra Ace™ qPCR RT Master Mix with gDNA Remover (TOYOBO, Osaka, Japan) as per the manufacturer's protocol. Quantitative real-time polymerase chain reaction (qRT-PCR) was performed on a CFX Connect™ Real-Time System (Bio-Rad, California, USA) using cDNA as the template, mixed with THUNDERBIRD™ Next SYBR® qPCR Mix (TOYOBO, Osaka, Japan). The cycling conditions were as follows: initial denaturation at 95 °C for 60 s, followed by 40 cycles of denaturation at 95 °C for 10 s, annealing at 61.1, 62.4, 64.9, and 66.1 °C for 15 s, and extension at 72 °C for 30 s. Each reaction was performed in triplicate to ensure reproducibility. Primer sequences used for the target and reference genes are listed in Supplementary Table S1. Gene expression was normalized to the housekeeping gene GAPDH, and relative fold changes in expression between treatment and control groups were calculated using the 2^−ΔΔCt^ method.

### 2.13 Enzyme-Linked Immunosorbent Assay (ELISA)

The secretion of collagen type I from HDFs treated with PBMC secretomes was quantified using the human collagen type I alpha 1 (COL1A1) ELISA Kit (ABclonal, Wuhan, China). HDFs were cultured in a 96-well plate and exposed to PBMC secretomes for a specified duration, after which the cell culture supernatants were collected for analysis. The ELISA procedure was conducted in accordance with the manufacturer's instructions to ensure accuracy and reproducibility.

### 2.14 Statistical analysis

The results data are presented as mean ± standard deviation (SD). Statistical differences among treatment groups were analyzed using one-way ANOVA followed by Tukey's multiple-comparison post-hoc test. Analyses were performed using OriginPro 2023 software (OriginLab, Northampton, MA, USA). A p-value of less than 0.05 was considered statistically significant.

## 3 Results

### 3.1 The secretome profile of IronQ-preconditioned PBMCs is enriched with trophic factors

**Fig. 2:**
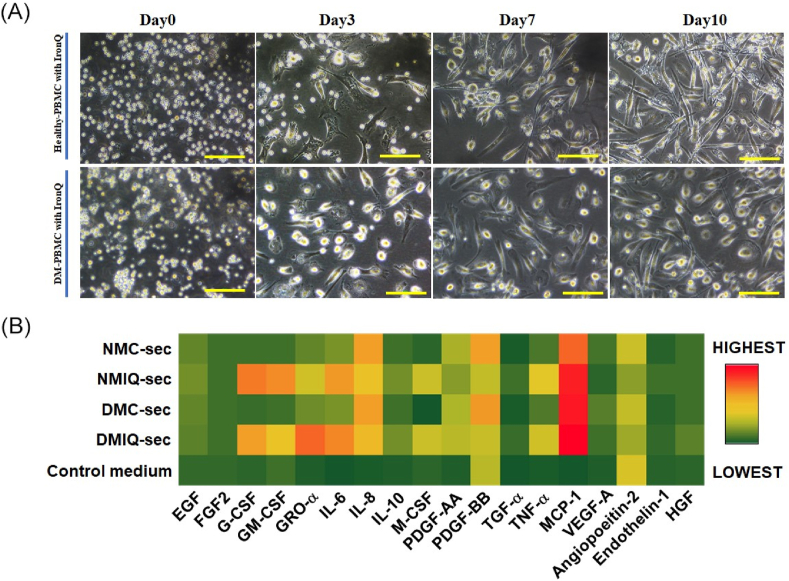

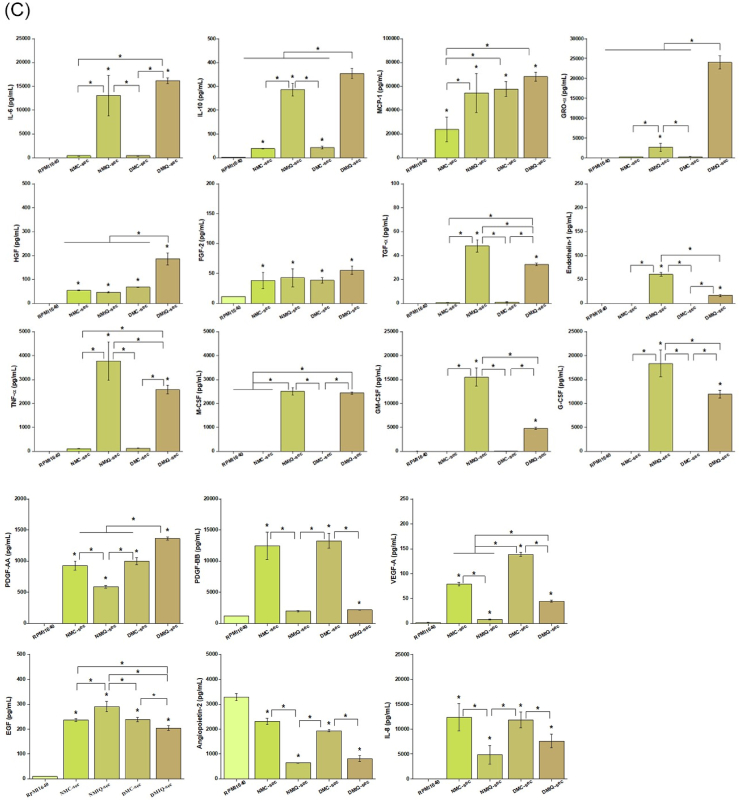

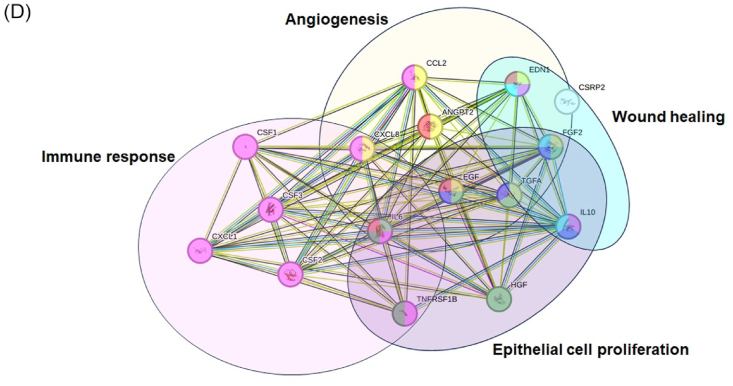
Characterization of soluble biofactors in IronQ-preconditioned PBMC secretomes. (A) Representative images showing the morphology of PBMCs treated with 125 μg/mL IronQ for 10 days. Scale bar, 100 μm (B) Heatmap illustrating the overall profile of 18 trophic factors, cytokines, and chemokines identified in PBMC secretomes, with protein abundance represented as mean values. Pooled secretomes from n = 10 donors per condition were analyzed. Each column corresponds to a specific protein, and each row represents a sample. Protein abundance is color-coded, ranging from dark green (low) to red (high). (C) Quantification of 18 soluble biofactors in NMC-sec, NMIQ-sec, DMC-sec, and DMIQ-sec, determined using Luminex multiplex technology. Data are expressed as mean ± SD in pg/mL, based on three independent experiments. ∗p < 0.05. (D) STRING analysis of protein-protein interaction (PPI) networks for the most abundant trophic factors expressed in PBMC secretomes, visualized using Cytoscape. Abbreviations: EGF, epidermal growth factor; FGF, fibroblast growth factor 2; HGF, hepatocyte growth factor; IL-6, interleukin 6; IL-8, interleukin 8; IL-10, interleukin 10; MCP-1, monocyte chemotactic protein 1; PDGF-AA, platelet-derived growth factor subunit A; PDGF-AB/BB, platelet-derived growth factor subunits A and B; VEGF-A, vascular endothelial growth factor-A; TGF-α, transforming growth factor alpha; TNF-α, tumor necrosis factor alpha; GRO-α, growth-regulated oncogene α; G-CSF, granulocyte colony-stimulating factor; GM-CSF, granulocyte-macrophage colony-stimulating factor; M-CSF, macrophage colony-stimulating factor.

To evaluate the effects of IronQ on PBMC secretome generation, we assessed changes in cell morphology, cell quality, and the protein composition of the secretome in PBMCs derived from healthy individuals (NMC-sec) and diabetic individuals (DMC-sec), comparing these to IronQ-preconditioned PBMCs from healthy (NMIQ-sec) and diabetic (DMIQ-sec) individuals. Under IronQ preconditioning for 10 days, PBMCs showed notable morphological changes, transforming into elongated, spindle-shaped cells with a gradual increase in cell confluence from Day 3 to Day 10. Importantly, no signs of cellular stress or morphological abnormalities were observed (Fig. 2A), suggesting that IronQ promotes cell growth while maintaining cellular integrity. To investigate IronQ's potential to enhance tissue repair processes, including wound healing and angiogenesis, we focused on analyzing 21 key factors within the secretome, which were selected for their known roles in cell proliferation, migration, angiogenesis, and epithelialization—key processes in wound healing and tissue regeneration. This selection ensured a comprehensive profile of the secretome's regenerative and therapeutic potential.

Using the MILLIPLEX® MAP Human Cytokine/Chemokine/Growth Factor Panel A and the MILLIPLEX MAP Human Angiogenesis/Growth Factor Magnetic Bead Panel, we identified 21 secreted factors across the four conditioned media (CMs), each displaying varying concentrations. Limited data were available for bone morphogenetic protein 9 (BMP-9), endoglin, and leptin, as these analytes were not detected in any of the CM conditions; therefore, they were excluded from the analysis. Temporal changes in expression levels were visualized through a heatmap (Fig. 2B), while a bar graph displayed the mean concentrations of the analytes, highlighting differences in expression levels (Fig. 2C).

Our analysis revealed that 61.9 % of factors were upregulated in IronQ-treated conditions (NMIQ-sec and DMIQ-sec) compared to non-treated conditions (NMC-sec and DMC-sec), with 19.05 % of factors downregulated and 19.05 % showing similar secretion levels. Among the most upregulated factors were those crucial for cell proliferation and migration, such as hepatocyte growth factor (HGF), fibroblast growth factor 2 (FGF2), epidermal growth factor (EGF), transforming growth factor alpha (TGF-α), and platelet-derived growth factor-AA (PDGF-AA). Additionally, factors involved in angiogenesis (HGF, EGF, FGF2, and IL-6), monocyte recruitment (monocyte chemoattractant protein-1 (MCP-1), GRO-α), immunomodulation, and monocyte-to-macrophage differentiation (G-CSF, M-CSF, GM-CSF, IL-10, and TNF-α) were significantly increased. Interestingly, PBMCs derived from diabetic individuals treated with IronQ (DMIQ-sec) demonstrated higher expressions of key factors like HGF, FGF2, PDGF-AA, and GRO-α compared to those from healthy individuals (NMIQ-sec). These factors are known for promoting keratinocyte proliferation, migration, and angiogenesis, essential components of re-epithelialization and wound healing. Notably, GRO-α was detected at significantly higher levels in DMIQ-sec (24053.13 pg/mL) compared to NMIQ-sec (2646.94 pg/mL) and pre-IronQ treatment samples (NMC-sec and DMC-sec at 231.87 and 268.88 pg/mL, respectively), indicating that IronQ may considerably enhance the regenerative potential of diabetic PBMCs. In addition, several factors, including Endothelin-1, TGF-α, M-CSF, G-CSF, IL-10, IL-6, and TNF-α, were significantly elevated in IronQ-treated samples. To further investigate the biological interactions of these secretome factors, we conducted a protein-protein interaction (PPI) network analysis using the STRING database. Gene ontology (GO) enrichment analysis revealed that the consistently abundant proteins in NMIQ-sec and DMIQ-sec samples play significant roles in angiogenesis, epithelial cell proliferation, cell migration, immune response, and wound healing (Fig. 2D). In summary, the broad spectrum of growth factors and cytokines in the IronQ preconditioned secretome indicates that IronQ treatment enhances the regenerative potential of PBMCs, especially in diabetic conditions where wound healing is typically impaired. These findings suggest that IronQ may be a valuable tool in improving the therapeutic efficacy of PBMCs for applications in wound repair and tissue regeneration.

### 3.2 The secretome profile of IronQ-preconditioned PBMCs analysis with ^1^H NMR

**Fig. 3:**
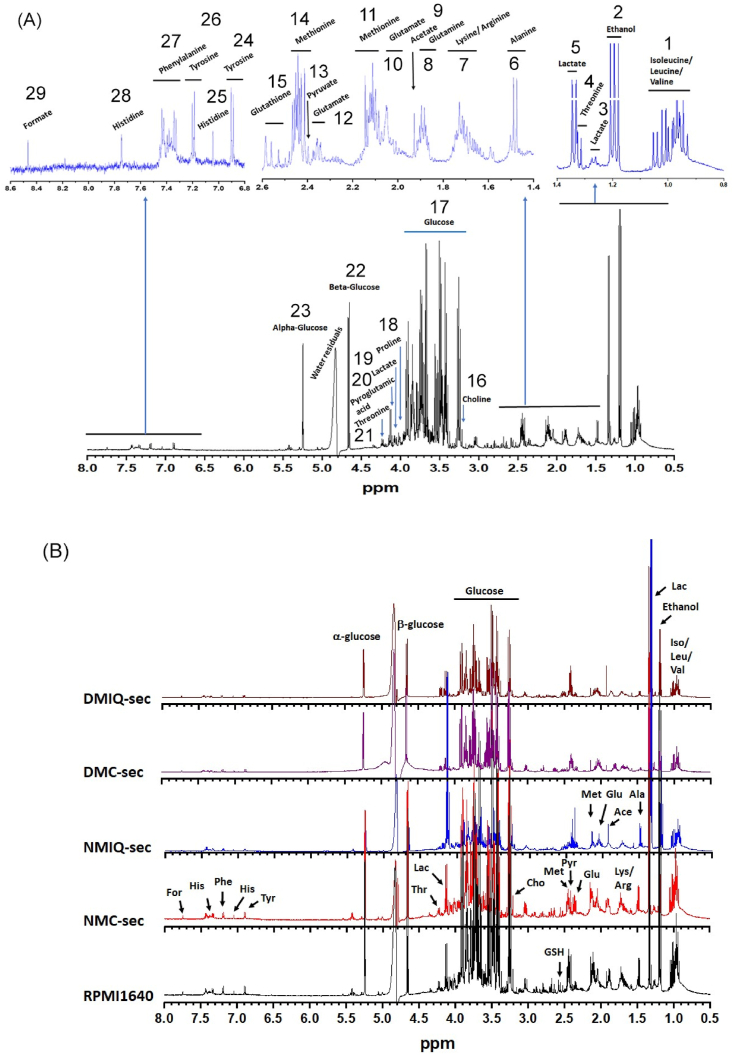
Representative ^1^H NMR spectra of PBMC secretomes recorded at 500 MHz. (A) Typical 1H NMR spectrum of PBMC secretomes, with characteristic metabolite signals labeled. The intensity of three spectral regions (0.8–1.4 ppm, 1.4–2.6 ppm, and 6.8–8.6 ppm) is magnified for better visualization. Numbers (1–29) correspond to metabolite assignments detailed in Supplementary Table S2. (B) Representative ^1^H NMR spectra of RPMI 1640 plain medium, NMC-sec, NMIQ-sec, DMC-sec, and DMIQ-sec.

Proton NMR spectroscopy was utilized to evaluate the metabolic changes in PBMC secretomes induced by IronQ preconditioning, based on the analysis of the conditioned media (CMs) produced by the cells. High-resolution ^1^H NMR spectra were acquired using optimized techniques to suppress solvent and other interfering signals. Consistent experimental conditions—including temperature, sample/solvent volumes, TSP concentration as an internal standard, and NMR acquisition and processing parameters—were maintained to minimize external variability and accurately identify specific metabolite variations. The assignment of proton resonances in the ^1^H NMR spectra and the characterization of metabolite compositions were performed through detailed spectral analysis. This analysis considered specific NMR parameters reflecting the structural characteristics of the metabolites. The assignments were validated by comparison with published literature [[24], [25], [26], [27], [28]], ensuring accurate identification. Representative ^1^H NMR spectra highlighting the major metabolites detected across experimental groups are shown in Fig. 3A, while a detailed assignment of resonance signals to metabolites is provided in Supplementary Table S2. Characteristic resonance signals for metabolites were identified in the ^1^H NMR spectra of unconditioned RPMI 1640 medium (plain), NMC-sec, NMIQ-sec, DMC-sec, and DMIQ-sec shown in Fig. 3B. Visual inspection of the spectra revealed similar overall profiles among the groups; however, distinct differences in metabolite concentrations were evident, reflecting variations in secretory activity and metabolic responses. The relative concentrations of metabolites in PBMC secretomes were quantified by integrating the intensity areas of specific proton resonance signals in the ^1^H NMR spectra. The relative concentrations, along with the chemical shifts and assignments of the signals used for quantification, are summarized in Supplementary Table S3.

**Fig. 4:**
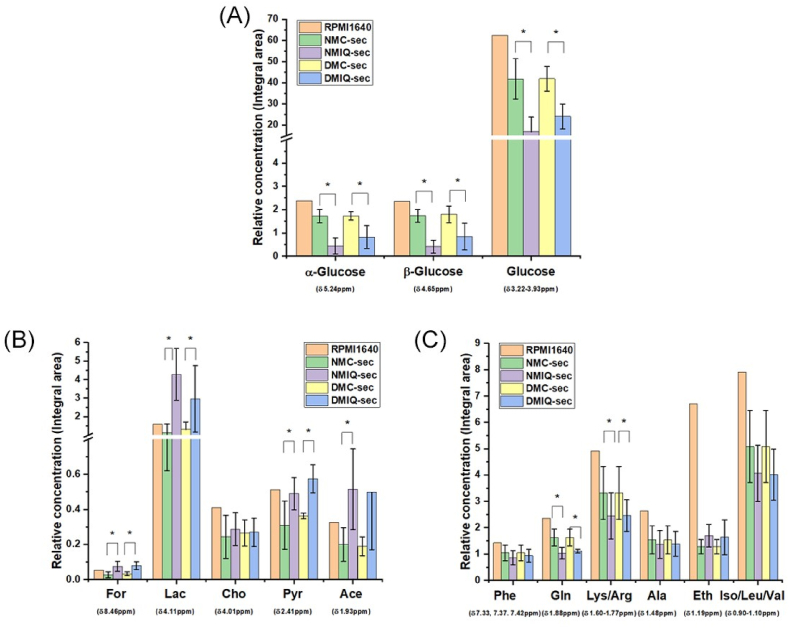
Graphical representation of relative metabolite concentrations in RPMI 1640 plain medium, NMC-sec, NMIQ-sec, DMC-sec, and DMIQ-sec. Bar graphs illustrate the relative concentrations of key metabolites as specific chemical shift (δ) detected across the experimental groups, highlighting differences in metabolite profiles between plain medium and PBMC secretomes derived from healthy (NMC-sec, NMIQ-sec) and diabetic (DMC-sec, DMIQ-sec) donors. Data are presented as mean ± SD from ten individual donors. ∗*p < 0.05* compared to non-conditioned PBMC secretome.

The ^1^H NMR spectra were dominated by proton resonance signals of glucose, the most abundant metabolite, alongside several low molecular weight compounds, including amino acids (alanine, tyrosine), organic acids (lactate, acetate, formate), and choline (Fig. 3B). Key metabolites, based on relative abundance, are visually highlighted in Fig. 4. Glucose appeared at the highest concentration in the spectra, with significantly higher levels in plain RPMI 1640 medium compared to PBMC-conditioned media. This observation aligns with glucose being a supplemented component of the RPMI 1640 medium. Both healthy and diabetic PBMCs exhibited substantial glucose consumption over the 10-day conditioning period, as indicated by its reduced levels in the conditioned media (Fig. 4A). Interestingly, pyruvate, a critical intermediate in glucose metabolism, was present in the spectra, along with its downstream catabolic products such as acetate, lactate, and ethanol. Notably, these metabolites displayed increased concentrations in IronQ-conditioned secretomes (NMIQ-sec and DMIQ-sec; Fig. 4B and C). Lactate levels were significantly elevated in IronQ-conditioned secretomes compared to their non-treated counterparts, indicating enhanced glycolytic activity. Similarly, acetate and formate levels were higher in NMIQ-sec and DMIQ-sec, suggesting increased metabolic activity and secretion in response to IronQ preconditioning. A comparative analysis of conditioned media (CMs) with unconditioned RPMI 1640 medium revealed two key trends: (1) a depletion of metabolites like glucose, reflecting cellular uptake and metabolism, and (2) an accumulation of secreted metabolites, including acetate, lactate, and formate, indicating active metabolic processing and excretion by PBMCs. These observations underscore the dynamic metabolic shifts occurring during the 10-day conditioning period. When comparing the metabolic profiles of secretomes derived from healthy donors (NMC-sec, NMIQ-sec) and diabetic donors (DMC-sec, DMIQ-sec), no statistically significant differences in metabolite concentrations were observed. However, subtle variations in profiles suggest a consistent metabolic response to IronQ treatment, independent of donor health status. These findings highlight the dual role of PBMCs during preconditioning: depleting metabolites from the medium through cellular uptake and actively secreting metabolites into the secretome. The observed increases in lactate, acetate, and formate concentrations in IronQ-conditioned secretomes strongly indicate that IronQ enhances metabolic activity and stimulates secretion, potentially contributing to the bioactivity of the PBMC secretome.

### 3.3 The secretome of IronQ pre-conditioning PBMCs promoted HUVEC proliferation, migration, and tube formation in vitro

**Fig. 5:**
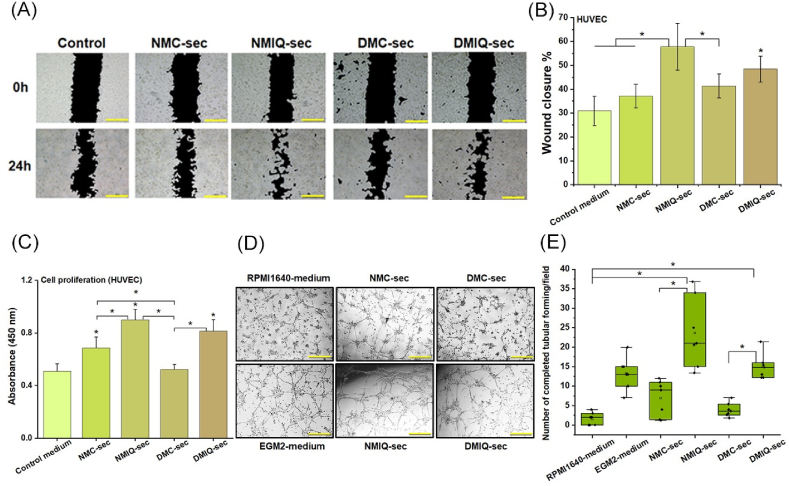
IronQ-preconditioned PBMC secretome enhances proliferation, migration, and tube formation in HUVECs. (A) Representative images from scratch wound healing assays showing HUVEC migration in the five experimental groups (Control, NMC-sec, NMIQ-sec, DMC-sec, and DMIQ-sec). And (B) quantitative analysis of the wound closure rate demonstrates enhanced migratory capacity in groups treated with IronQ-preconditioned PBMC secretomes (NMIQ-sec and DMIQ-sec) compared to the control group. Data are expressed as mean ± SD from three independent experiments. ∗*p < 0.05* versus the control group. (C) The proliferative effect of the IronQ-preconditioned PBMC secretome on HUVEC was evaluated using a CCK-8 assay after 72 h of treatment. Data are presented as mean ± SD from three independent experiments. ∗*p < 0.05* versus the control group. (D) Representative images from tube formation assays showing the ability of HUVECs to form capillary-like structures under different conditions. Scale bar, 500 μm. And (E) quantitative analysis of tubular structures per field reveals significantly increased tube formation in the NMIQ-sec and DMIQ-sec groups compared to the control group. Data are expressed as mean ± SD from three independent experiments. ∗*p < 0.05* versus the control group.

Endothelial cell proliferation, migration, and tube formation are essential steps in angiogenesis, a critical process in wound healing and tissue regeneration. To evaluate the effects of the secretome from IronQ-preconditioned PBMCs on endothelial cell behavior, we conducted in vitro assays using HUVECs. Specifically, we performed CCK-8 proliferation assays, scratch wound migration assays, and tube formation assays to assess HUVEC proliferation, migration, and capillary-like structure formation. The results showed that both the secretome from IronQ-preconditioned healthy PBMCs (NMIQ-sec) and diabetic PBMCs (DMIQ-sec) significantly enhanced HUVEC migration compared to untreated PBMC secretomes (NMC-sec, DMC-sec) and control medium. Notably, NMIQ-sec and DMIQ-sec exhibited similar capabilities to induce HUVEC migration, with DMIQ-sec demonstrating a slightly greater migratory effect than DMC-sec. This suggests that IronQ preconditioning positively influences the functional properties of PBMCs even under diabetic conditions, potentially compensating for compromised cellular functions associated with diabetes (Fig. 5A and B). In terms of proliferation, HUVECs treated with NMIQ-sec and DMIQ-sec showed a marked increase in proliferation compared to NMC-sec, DMC-sec, and control medium, underscoring the enhanced mitogenic potential of the IronQ-preconditioned secretome (Fig. 5C). These results highlight IronQ's role in stimulating factors that promote endothelial cell growth, which is critical for forming new blood vessels and supporting tissue repair. To assess capillary-like structure formation, we performed tube formation assays on Matrigel. The results demonstrated that both NMIQ-sec and DMIQ-sec significantly promoted tube formation, characterized by an increased number of interconnected tubes and more extensive, complete tubular networks compared to the control group. While DMC-sec alone exhibited limited tube formation capability, the secretome from IronQ-treated diabetic PBMCs (DMIQ-sec) significantly improved tube formation in HUVECs, indicating IronQ's ability to restore or enhance angiogenic function in diabetic PBMCs (Fig. 5D and E). Interestingly, RPMI 1640 medium alone, without the PBMC secretome, did not promote tube formation in HUVECs. This suggests that the pro-angiogenic effects are specifically mediated by the enhanced secretome from IronQ-preconditioned PBMCs, rather than from RPMI1640 medium itself. The enriched secretome likely contains a combination of growth factors, cytokines, and chemokines that collectively drive endothelial cell proliferation, migration, and tube formation. The findings indicate that the secretome of IronQ-preconditioned PBMCs plays a crucial role in promoting endothelial cell functions that are vital for angiogenesis.

### 3.4 The secretome of IronQ pre-conditioning PBMCs promotes proliferative and migratory abilities of dermal fibroblasts and keratinocytes

**Fig. 6:**
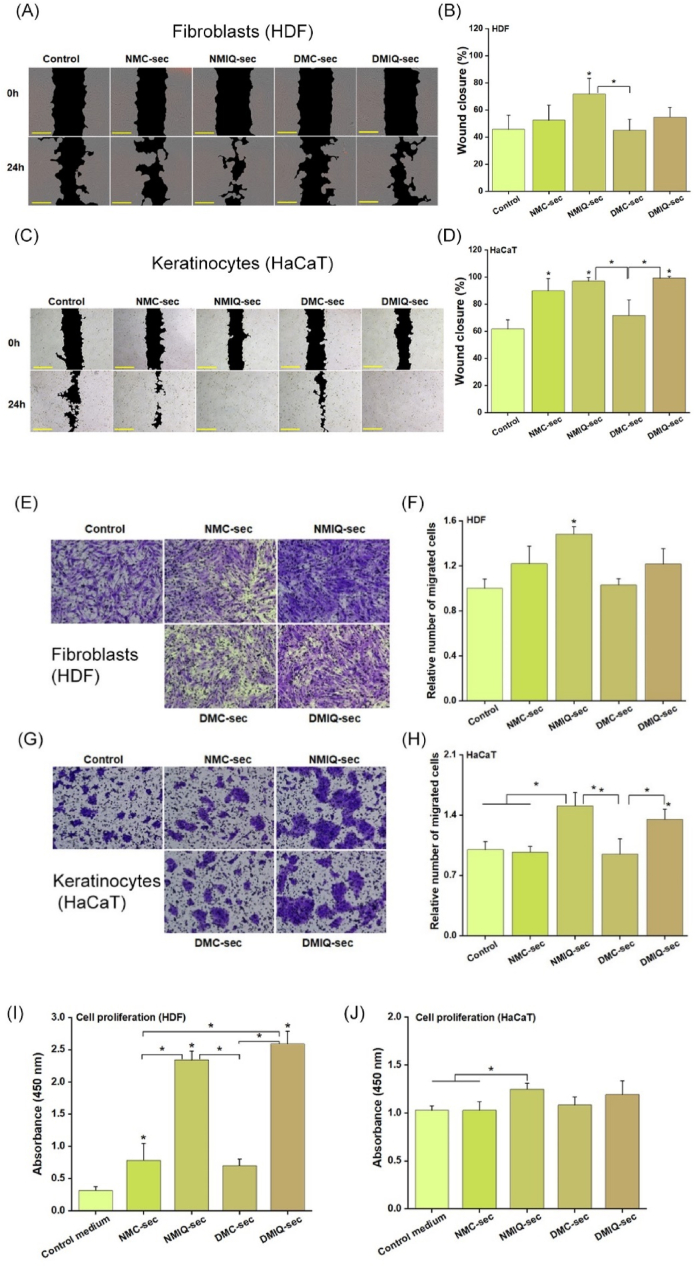
IronQ-preconditioned PBMC secretome enhances proliferation and migration of dermal Fibroblasts and Keratinocytes. (A, B) Representative images and quantitative analysis of fibroblast migration, assessed via a scratch assay. Scale bar, 500 μm. (C, D) Representative images and quantitative analysis of keratinocyte migration, assessed via a scratch assay. Scale bar, 500 μm. (E, F) Representative images and corresponding quantitative analysis of invaded fibroblasts, evaluated using a Transwell assay. Scale bar, 500 μm. (G, H) Representative images and corresponding quantitative analysis of invaded keratinocytes, evaluated using a Transwell assay. Scale bar, 500 μm. (I, J) The effect of the IronQ-preconditioned PBMC secretome on fibroblast and keratinocyte cell proliferation was measured using a CCK-8 assay after 72 h of treatment. Data are presented as mean ± SD from three independent experiments. ∗*p < 0.05*.

The proliferation and migration of dermal cellular components, such as fibroblasts and keratinocytes, are essential for effective cutaneous repair and regeneration. To investigate how the PBMC secretome contributes to wound healing, we assessed its impact on the proliferative and migratory abilities of these key dermal cell types. Our findings showed that exposure to the secretomes from IronQ-preconditioned PBMCs, derived from both healthy individuals (NMIQ-sec) and diabetic individuals (DMIQ-sec), significantly enhanced the migration of human dermal fibroblast cells and keratinocyte cells (Fig. 6A, B and Fig. 6C, D, respectively) as well as the invasion of these cells (Fig. 6E,F and Fig. 6G, H, respectively) compared to cells treated with non-preconditioned PBMC secretomes (NMC-sec, DMC-sec) and the control medium. This enhancement suggests that the trophic factors secreted by PBMCs in response to IronQ preconditioning play a critical role in facilitating these processes. Additionally, we evaluated the effect of the IronQ-preconditioned PBMC secretome on cell proliferation using the CCK-8 assay over a 3-day period in vitro. Treatment with both NMIQ-sec and DMIQ-sec significantly enhanced the proliferation of human dermal fibroblast cells and keratinocyte cells compared to cells treated with non-preconditioned PBMC secretomes (NMC-sec, DMC-sec) and the control medium, highlighting the positive influence of the IronQ-preconditioned secretome on cellular growth (Fig. 6I and J, respectively). These findings suggest that the secretome from IronQ-preconditioned PBMCs accelerates wound healing by significantly enhancing the proliferative and migratory capabilities of dermal cells. This improved cellular response highlights the potential of IronQ-preconditioned PBMCs as a therapeutic approach for promoting effective wound repair and tissue regeneration, particularly in cases where these processes may be impaired.

### 3.5 Treatment with the secretome of IronQ-preconditioned PBMCs enhances collagen expression and secretion by fibroblasts

**Fig. 7:**
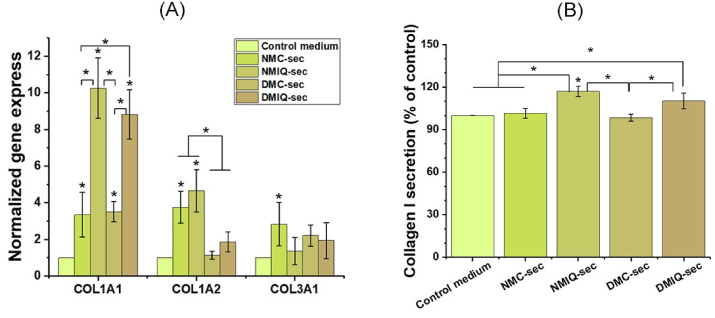
IronQ-preconditioned PBMC secretomes enhance collagen production in fibroblasts. (A) The relative mRNA expression levels of collagen type I alpha 1 chain (COL1A1), collagen type I alpha 2 chain (COL1A2), and collagen type III alpha 1 chain (COL3A1) in fibroblasts treated with PBMC secretomes were analyzed using qRT-PCR. Gene expression levels were normalized to the housekeeping gene (GAPDH) and quantified using the 2 ^−ΔΔCt^ method. Data are presented as mean ± SD from three independent experiments. (B) The secretion level of collagen types I of fibroblasts treated with PBMC secretomes was quantified in culture supernatants using ELISA. Data are expressed as the median values of duplicate samples from independent experiments.

To assess the impact of the secretome from IronQ-preconditioned PBMCs on collagen production in fibroblast cells, we measured collagen expression at mRNA levels. As shown in Fig. 7A, qRT-PCR analysis demonstrated a significant upregulation of collagen type I alpha 1 chain (COL1A1) and collagen type I alpha 2 chain (COL1A2) mRNA expression in fibroblasts treated with the secretome from IronQ-preconditioned PBMCs from both healthy individuals (NMIQ-sec) and diabetic individuals (DMIQ-sec), compared to fibroblasts treated with non-preconditioned PBMC secretomes (NMC-sec, DMC-sec) and the control medium. Notably, the increase in COL1A1 and COL1A2 expression was substantially greater than that of collagen type III alpha 1 chain (COL3A1), which exhibited minimal changes and even a slight decrease in the NMIQ-sec and DMIQ-sec treatment groups. To further evaluate collagen production at the protein level, we measured the collagen content in the supernatants of all treatment groups. Fibroblasts treated with the secretomes from NMIQ-sec and DMIQ-sec showed significantly higher levels of collagen type I (Col-1) protein compared to those treated with non-preconditioned PBMC secretomes (NMC-sec and DMC-sec) and the control medium (Fig. 7B). In contrast, the production of collagen type III (Col-3) protein remained relatively unchanged across these groups (Supplementary Fig. S1). This finding suggests that the IronQ-preconditioned secretome specifically enhances collagen type I production, the primary structural collagen involved in wound healing and maintaining tissue integrity. These findings highlight the potential of IronQ-preconditioned PBMC secretome as a therapeutic agent to support fibroblast-mediated tissue regeneration and enhance extracellular matrix remodeling, particularly in conditions where collagen synthesis is compromised.

## 4 Discussion

In this study, we analyzed the IronQ-preconditioned PBMC secretome using ^1^H NMR and MILLIPLEX® Multiplex ELISA assay, revealing an enriched profile of growth factors, chemokines, and cytokines, positioning it as a promising source for autologous, cell-free therapies in diabetic wound healing. Key factors—including Endothelin-1, TGF-α, G-CSF, GM-CSF, MCP-1, TNF-α, IL-6, and IL-10—were present at elevated levels in the IronQ-preconditioned PBMC secretome. Mechanistically, multiple factors enriched in the IronQ-preconditioned PBMC secretome converge on the core programs of repair. Endothelin-1 and TGF-α stimulate keratinocyte and fibroblast proliferation, collagen deposition, and angiogenesis; TGF-α also enhances keratinocyte migration to drive re-epithelialization, and in combination with PDGF-BB accelerates closure in diabetic mice [39,40]. G-CSF, M-CSF, and GM-CSF support hematopoiesis, mobilize progenitors, and modulate innate immunity; notably, G-/GM-CSF promote endothelial proliferation, and G-CSF recruits pro-angiogenic cells via the SDF-1/CXCR4 axis—effects that improve neovascularization and wound closure, including in diabetic models and when paired with AMD3100 [[41], [42], [43]]. IL-10 adds an anti-inflammatory tone while fostering EPC recruitment and re-epithelialization. The concurrent presence of TNF-α and IL-6 indicates a coordinated pro-healing milieu coupling early inflammation with angiogenesis; IL-6 is pleiotropic—chemotactic for macrophages, supportive of the M1→M2 transition, stimulatory for fibroblast proliferation/migration, and an inducer of VEGF to drive endothelial growth and tubulogenesis—consistent with delayed angiogenesis and reduced VEGF in IL-6 knockout wounds [[44], [45], [46], [47], [48]]. Notably, IronQ-preconditioned PBMCs from diabetic donors released higher HGF, PDGF-AA, and GRO-α than those from healthy donors. HGF suppresses excessive inflammation, promotes granulation and angiogenesis, and accelerates re-epithelialization via β1-integrin/ILK signaling; exogenous HGF improves healing in diabetic mice [[49], [50], [51], [52]]. PDGF-AA advances fibroblast proliferation/migration, pericyte recruitment, and matrix deposition, stabilizing nascent vessels. GRO-α/CXCL1 is strongly mitogenic for keratinocytes and enhances re-epithelialization in vivo while tempering contraction, favoring balanced closure [53,54]. Together, these selectively enriched mediators suggest that IronQ conditioning “tunes” PBMC output to counter diabetic deficits—enhancing angiogenesis, epithelial repair, and perfusion of metabolically compromised wound beds.

The ^1^H NMR profiling of conditioned media (CM) illuminated how PBMC metabolism adapts during IronQ exposure. Glucose dominated the spectra—consistent with its central role in energy supply—but the overall signature indicated PBMCs did not rely solely on glycolysis for ATP generation. Although detected at low concentrations, pyruvate showed modest time-dependent utilization, highlighting its role as a metabolic hub feeding multiple pathways [55]. Its relatively stable levels despite use suggest ongoing endogenous regeneration through non-glucose-dependent biosynthetic routes [56]. A progressive rise in acetate—an intermediate of oxidative pyruvate catabolism—supports flux toward acetyl-CoA and the TCA cycle. At the same time, elevated choline and formate (which intersect one-carbon and acetyl-CoA/acetate metabolism) further point to a predominance of oxidative pathways. Concomitantly, increased lactate in CM indicates parallel anaerobic flux from pyruvate, consistent with glycolytic compensation under higher energetic demand or localized oxygen limitation [57]. Together, these readouts reveal marked metabolic flexibility: PBMCs engage both aerobic (TCA/oxidative) and anaerobic (glycolytic/fermentative) routes to sustain ATP production and precursor pools. This balanced bioenergetic strategy likely supports cell survival and the biosynthesis of cytokines, chemokines, and growth factors, enriching the secretome. Overall, the metabolic profile is consistent with IronQ preconditioning that reprograms PBMC metabolism toward a more pro-regenerative, bioactive secretory output.

After profiling the secretome, we tested its biological activity in vitro, focusing on angiogenesis, which is central to tissue regeneration and wound repair. Both IronQ-preconditioned secretomes (NMIQ-sec and DMIQ-sec) significantly enhanced HUVEC proliferation, tube formation, and (to a lesser extent) migration compared with their non-preconditioned counterparts (NMC-sec and DMC-sec). Notably, the most significant gain was in tube formation, whereas migration showed no significant additional improvement beyond baseline PBMC secretome effects. This pattern suggests that IronQ preconditioning preferentially augments factors that drive endothelial organization and lumenization rather than chemotactic cues per se. Consistent with this, MCP-1—a key mitogen for endothelial cells—was already abundant in PBMC secretomes and increased only modestly with IronQ [58], implying that the superior tube-forming activity is more likely attributable to other pro-angiogenic mediators enriched by IronQ (e.g., HGF, PDGF, IL-6/TNF-linked VEGF induction) rather than differential MCP-1 signaling. We next assessed whether IronQ-preconditioned PBMC secretomes could accelerate key cellular programs of cutaneous repair. In normal healing, keratinocytes proliferate and migrate from the wound edge to re-establish barrier function, dermal fibroblasts infiltrate the wound bed to deposit and remodel extracellular matrix, and endothelial cells form new microvasculature to restore oxygen and nutrient delivery [59,60]. These processes are blunted in chronic wounds, where reduced proliferation and migration impede closure [61]. In our assays, IronQ-preconditioned secretomes significantly increased fibroblast, keratinocyte, and endothelial invasion/migration and proliferation versus non-preconditioned controls. They also enhanced collagen synthesis—particularly types I and III—central ECM components that confer early scaffold (type III) and later tensile strength (type I) during maturation [62]. A shift toward a higher type I: type III ratio is consistent with matrix maturation and improved biomechanical integrity, features linked to more efficient wound resolution [63]. Although IronQ-conditioned PBMC factors increased fibroblast motility and growth in vitro, pathological scarring typically reflects sustained profibrotic signaling rather than transient repair cues. The unchanged type III collagen (Col-III) under our dosing paradigm argues against a persistently fibrogenic state. Consistent with prior reports, secretome-based interventions can accelerate repair without promoting fibrosis [64,65]. Nevertheless, because fibrosis is a tissue-level phenomenon, future in vivo studies will use dose- and time-limited IronQ exposure and explicitly assess hypertrophic-scar endpoints alongside a profibrotic marker panel. Together, these data indicate that IronQ preconditioning amplifies the pro-regenerative capacity of the PBMC secretome across multiple effector cell types, supporting a cell-free strategy to hasten wound repair and improve outcomes in chronic and diabetic wounds.

Notably, the angiogenic and tissue-repair activities of IronQ-preconditioned PBMC secretomes did not differ significantly between healthy and diabetic donors. This aligns with our prior work showing that IronQ-treated PBMCs from both groups exhibit comparable expression of angiogenic and regenerative gene programs, supporting IronQ preconditioning as a viable autologous strategy for diabetic patients. Several limitations warrant attention. The sample size was modest, and efficacy was assessed in vitro; next steps include validation in relevant in vivo wound models, followed by early-phase clinical trials to evaluate safety, dosing, delivery format, and durability of benefit. Future studies should also enroll more diverse cohorts to ensure generalizability across age, sex, ethnicity, and disease severity. Sex as a biological variable: Nevertheless, the current study used male donors only to minimize variability from cyclical ovarian hormones that can influence innate-immune readouts and potentially obscure IronQ-specific effects. We acknowledge this limits generalizability and will address it by including female donors with menstrual-phase stratification (or hormone measurements as covariates) and powering sex-stratified analyses to test whether IronQ's pro-regenerative actions differ by sex rigorously. Finally, to our knowledge, this work represents the first preclinical evaluation of IronQ-preconditioned PBMC products derived from diabetic blood, establishing feasibility and laying the groundwork for larger, multi-center studies.

In conclusion, IronQ-preconditioned PBMC secretomes emerge as a promising, cell-free therapy for diabetic wound healing. IronQ tuning enriches the PBMC secretory profile with pro-angiogenic and immunomodulatory mediators (e.g., TGF-α, TNF-α, IL-6, IL-10, HGF, G-CSF, M-CSF, GM-CSF, GRO-α), thereby addressing hallmark deficits of diabetic wounds in angiogenesis, inflammation control, and cellular function. Functionally, these secretomes enhanced endothelial proliferation and tube formation, stimulated fibroblast and keratinocyte migration, and increased collagen synthesis—processes essential for effective repair. Metabolomic readouts indicate that PBMCs maintain aerobic and anaerobic energy pathways under IronQ exposure, supporting sustained biosynthesis of bioactive factors. Notably, therapeutic activity was comparable between secretomes derived from healthy and diabetic donors, underscoring the feasibility of an autologous approach in diabetes. Given their scalability, reduced immunogenic risk, and manufacturing tractability, IronQ-preconditioned secretomes warrant further development. Next steps include validation in relevant in vivo diabetic wound models, assessment of safety, dosing, and durability, and evaluation across diverse patient populations to support clinical translation of this cost-effective, minimally invasive strategy for chronic and diabetic wounds.

## Funding sources

This study was supported by the Faculty of Associated Medical Sciences, Chiang Mai University, Chiang Mai, Thailand (Grant Number R65IN00026), with partial financial support from the Franco-Thai Cooperation Programme in Higher Education and Research/Franco-Thai Mobility Programme/PHC SIAM 2022–2023 and Chiang Mai University, Thailand (Grant No. PM09/2566).

## CRediT authorship contribution statement

**Jiraporn Kantapan:** Conceptualization, Data curation, Formal analysis, Funding acquisition, Investigation, Methodology, Resources, Validation, Visualization, Writing – original draft. **Phattarawadee Innuan:** Formal analysis, Investigation, Writing – original draft. **Chonticha Sirikul:** Formal analysis, Investigation, Writing – original draft. **Nampeung Anukul:** Data curation, Resources, Validation, Writing – review & editing. **Gwenaël Rolin:** Data curation, Funding acquisition, Methodology, Resources, Validation, Writing – review & editing. **Nathupakorn Dechsupa:** Conceptualization, Data curation, Formal analysis, Funding acquisition, Methodology, Project administration, Resources, Supervision, Validation, Writing – review & editing.

## Declaration of competing interest

The authors declare the following financial interests/personal relationships which may be considered as potential competing interests: Jiraporn Kantapan reports financial support was provided by 10.13039/501100002842Chiang Mai University. If there are other authors, they declare that they have no known competing financial interests or personal relationships that could have appeared to influence the work reported in this paper.

## Data Availability

Data will be made available on request.
