# Supplementary material for: Characterization of PBMC secretome: Iron-quercetin preconditioning enhances pro-angiogenic and tissue regeneration factors for potential autologous diabetic wound healing applications
Source: Biochem Biophys Rep. 2025 Nov 5;44:102338. doi: 10.1016/j.bbrep.2025.102338 (PMC12636337; doi:10.1016/j.bbrep.2025.102338)
Supplement: Multimedia component 1 [file mmc1.docx]

Characterization of PBMC secretome: Iron-quercetin precondi-tioning enhances pro-angiogenic and tissue regeneration factors for potential autologous diabetic wound healing applications

Jiraporn Kantapan ^1,2^, Phattarawadee Innuan ^1,2^, Chonticha Sirikul ^3^, Nampeung Anukul ^3^, Gwenaël Rolin ^4,5^, Nathupakorn Dechsupa ^1,2^ *

^1^ Molecular Imaging and Therapy Research Unit, Department of Radiologic Technology, Faculty of Associated Medical Sciences, Chiang Mai University, Chiang Mai 50200, Thailand; [jiraporn.kan@cmu.ac.th](mailto:jiraporn.kan@cmu.ac.th), [phattarawadee_in@cmu.ac.th](mailto:phattarawadee_in@cmu.ac.th), [nathupakorn.d@cmu.ac.th](mailto:nathupakorn.d@cmu.ac.th)

^2^ Department of Radiologic Technology, Faculty of Associated Medical Sciences, Chiang Mai University, Chiang Mai 50200, Thailand; [jiraporn.kan@cmu.ac.th](mailto:jiraporn.kan@cmu.ac.th), [phattarawadee_in@cmu.ac.th](mailto:phattarawadee_in@cmu.ac.th), [nathupakorn.d@cmu.ac.th](mailto:nathupakorn.d@cmu.ac.th)

^3^ Division of Transfusion Science, Department of Medical Technology, Faculty of Associated Medical Sciences, Chiang Mai University, Chiang Mai 50200, Thailand; [chonthicha.sir@cmu.ac.th](mailto:chonthicha.sir@cmu.ac.th), [nampeung.a@cmu.ac.th](mailto:nampeung.a@cmu.ac.th)

^4^ INSERM CIC-1431, CHU Besançon, F-25000 Besançon, France; [gwenael.rolin@univ-fcomte.fr](mailto:gwenael.rolin@univ-fcomte.fr)

^5^ Université Marie et Louis Pasteur, UM RIGHT, F-25000 Besançon, France; [gwenael.rolin@univ-fcomte.fr](mailto:gwenael.rolin@univ-fcomte.fr)

* Correspondence: [nathupakorn.d@cmu.ac.th](mailto:nathupakorn.d@cmu.ac.th); Tel.: 66-53949214

**Supplementary Table**

**Supplementary Table S1.** List of primers used in quantitative real-time polymerase chain reaction (qRT-PCR).

| Target | Forward Primer (5’ to 3’) | Reverse Primer (5’ to 3’) |
| --- | --- | --- |
| *COL1A1* | CTGGTGATGCTGGTGCTAAAG | GACCTTTGCCGCCTTCTTTGC |
| *COL1A2* | GTGGTGACCAAGGTCCAGTTG | GCCAAGAGGACCAGGTTCAC |
| *COL3A1* | GAGCTAAAGGCGAAGATGGC | GCCATCTCTGCCAGGTTCTC |
| *GAPDH* | GTATCGTGGGAAGGACTCATGAC | GAACATCATCCCTGCCTCTAC |

**Supplementary Table S2.** ^1^ H NMR chemical shifts and multiplicity of the main metabolites observed in the spectra of plain medium (RPMI 1640) and PBMC secretomes.

| **No.** | **Metabolites** | **Abbreviation** | **MW (g/mol)** | **Group** | **Number of resonance proton** | **Chemical shift,**  **ppm (multiplicity)** |
| --- | --- | --- | --- | --- | --- | --- |
| 1 | Isoleucine/Leucine/Valine | Iso/Leu/Val | 379.5 | CH3 | 18 | 0.9-1.1 |
| 2 | Ethanol | Eth | 46.1 | CH3 | 3 | 1.19 (t) |
| 3 | Lactate | Lac | 89.1 | CH3 | 3 | 1.25 (d) |
| 4 | Threonine | Thr | 119.1 | gCH3 | 3 | 1.31(d) |
| 5 | Lactate | Lac | 89.1 | bCH3 | 3 | 1.33(d) |
| 6 | Alanine | Ala | 89.1 | bCH3 | 3 | 1.48 (d) |
| 7 | Lysine/Arginine | Lys/Arg | 320.4 | CH/CH2 | 3 | 1.60-1.77 (m) |
| 8 | Glutamine | Glu | 146.2 | bCH2 | 2 | 1.88 (m) |
| 9 | Acetate | Ace | 59 | CH3 | 3 | 1.93 (s) |
| 10 | Glutamate | Glu | 147.1 | bCH2 | 2 | 2.05 (m) |
| 11 | Methionine | Met | 131.2 | bCH2 | 2 | 2.11 (m) |
| 12 | Glutamate | Glu | 147.1 | gCH2 | 2 | 2.35 (m) |
| 13 | Pyruvate | Pyr | 87.5 | CH3 | 3 | 2.41 (s) |
| 14 | Methionine | Met | 131.2 | gCH2 | 2 | 2.45 (m) |
| 15 | Glutathione | GSH | 307.3 | CH2 | 2 | 2.56 (m) |
| 16 | Choline | Cho | 104.2 | NCH3 | 3 | 3.21 (s) |
| 17 | Glucose | Glucose | 180.1 | C6H12O6 | 12 | 3.22-3.93 (m) |
| 18 | Proline | Pro | 115.1 | H10 | 1 | 4.01 (m) |
| 19 | Lactate | Lac | 89.1 | aCH | 1 | 4.11 (q) |
| 20 | Pyroglutamic acid | Pyr | 129.1 | CH | 1 | 4.19 (q) |
| 21 | Threonine | Thr | 119.1 | bCH2 | 2 | 4.26 (m) 4.34 (m) |
| 22 | beta-Glucose | β-Glucose | 180.1 | H1 | 1 | 4.65 (d) |
| 23 | alpha-Glucose | α-Glucose | 180.1 | H1 | 1 | 5.24 (d) |
| 24 | Tyrosine | Tyr | 163.2 | H3,5 | 2 | 6.89 (d) |
| 25 | Histidine | His | 155.2 | H5 | 1 | 7.04 (s) |
| 26 | Tyrosine | Tyr | 163.2 | H2,6 | 2 | 7.19 (d) |
| 27 | Phenylalanine | Phe | 147.2 | H4 | 1 | 7.33, 7.37, 7.42 (m) |
| 28 | Histidine | His | 155.2 | H5 | 1 | 7.74 (s) |
| 29 | Formate | For | 45 | CH | 1 | 8.46 (s) |

**Supplementary Table S3. Relative metabolite concentration (integral area).** Calculated from ^1^ H-NMR spectra of unconditioned (Plain Medium) and PBMC secretome (NMC-sec, NMIQ-sec, DMC-sec, and DMIQ-sec). Data presented in Mean ± (SD).

| **No.** | **Metabolites** | δ, **ppm (multiplicity)** | **Relative concentration (Integral area)** | | | | |
| --- | --- | --- | --- | --- | --- | --- | --- |
|  |  |  | **RPMI1640** | **NMC-sec**  **(SD)**  **N=10** | **NMIQ-sec**  **(SD)**  **N =10** | **DMC-sec (SD)**  **N = 10** | **DMIQ-sec (SD)**  **N =10** |
| 1 | Isoleucine/ Leucine/ Valine | 0.9-1.1 | 7.90857 | 3.915962  (2.771764) | 3.144071  (2.015598) | 2.606334  (2.487162) | 3.460886  (1.858263) |
| 2 | Ethanol | 1.19 (t) | 6.70666 | 1.348927  (0.397739) | 1.696779  (0.43009) | 1.276262  (0.269455) | 1.63979  (0.661512) |
| 3 | Lactate | 1.25 (d) | 1.22694 | 0.818003  (0.29501) | 0.502915  (0.147902) | 0.830591  (0.201896) | 0.601687  (0.163298) |
| 4 | Threonine | 1.31(d) | 0.532464 | 0.33879  (0.132283) | 0.165457  (0.054136) | 0.340925  (0.126368) | 0.302933  (0.055213) |
| 5 | Lactate | 1.33(d) | 3.55584 | 3.132505  (0.643204) | 13.17451  (4.356569) | 3.148933  (1.199564) | 8.960663  (5.779977) |
| 6 | Alanine | 1.48 (d) | 2.64549 | 1.428613  (0.577372) | 1.361544  (0.525813) | 1.535574  (0.532472) | 1.384591  (0.474111) |
| 7 | Lysine/Arginine | 1.60-1.77 (m) | 4.9083 | 3.479983  (1.0584) | 2.448506  (0.884504) | 3.321054  (1.006937) | 2.461641  (0.597564) |
| 8 | Glutamine | 1.88 (m) | 2.35184 | 1.561033  (0.455572) | 1.026  (0.22011) | 1.62305  (0.322476) | 1.112326  (0.074204) |
| 9 | Acetate | 1.93 (s) | 0.325067 | 0.204743  (0.086844) | 1.492462  (1.673078) | 0.189751  (0.0541) | 1.109191  (1.010824) |
| 10 | Glutamate | 2.05 (m) | 3.50861 | 2.231376  (0.687664) | 2.213926  (0.852694) | 2.572057  (0.61178) | 2.213978  (0.657485) |
| 11 | Methionine | 2.11 (m) | 3.99144 | 2.334607  (0.674394) | 1.350005  (0.391488) | 2.136427  (0.773444) | 1.470645  (0.34618) |
| 12 | Glutamate | 2.35 (m) | 1.46218 | 1.313558  (0.393877) | 1.282594  (0.276906) | 1.144807  (0.295232) | 1.174338  (0.342637) |
| 13 | Pyruvate | 2.41(s) | 0.511154 | 0.371078  (0.08228) | 0.489643  (0.091351) | 0.363201  (0.016382) | 0.57531  (0.079948) |
| 14 | Methionine | 2.45 (m) | 2.56443 | 1.647831  (0.397835) | 1.235788  (0.251183) | 1.736897  (0.559416) | 1.267001  (0.457076) |
| 15 | Glutathione | 2.56 (m) | 0.384671 | 0.285288  (0.155502) | 0.192144  (0.077105) | 0.286475  (0.207188) | 0.181789  (0.074189) |
| 16 | Choline | 3.21 (s) | 0.409531 | 0.300445  (0.107308) | 0.286606  (0.094158) | 0.265528  (0.075458) | 0.269593  (0.0815) |
| 17 | Glucose | 3.22-3.93 (m) | 62.3811 | 41.77063  (9.484812) | 16.87334  (6.815339) | 41.84687  (5.857409) | 23.99313  (5.930065) |
| 18 | Proline | 4.01 (m) | 1.06883 | 1.051689  (0.373561) | 0.841077  (0.357812) | 0.938308  (0.429837) | 0.754352  (0.359407) |
| 19 | Lactate | 4.11 (q) | 1.5877 | 1.358336  (0.334101) | 4.274287  (1.403917) | 1.321433  (0.396962) | 2.96169  (1.786238) |
| 20 | Pyroglutamic acid | 4.19 (q) | 0.604969 | 0.451167  (0.134804) | 0.590497  (0.164887) | 0.467471  (0.060682) | 0.673944  (0.136999) |
| 21 | Threonine | 4.26 (m) 4.34 (m) | 0.219349 | 0.176727  (0.059586) | 0.160955  (0.215082) | 0.161021  (0.053494) | 0.11727  (0.063291) |
| 22 | beta-Glucose | 4.65 (d) | 2.3551 | 1.738631  (0.275384) | 0.411367  (0.278903) | 1.79506  (0.355287) | 0.841425  (0.5723) |
| 23 | alpha-Glucose | 5.24 (d) | 2.38034 | 1.718745  (0.285163) | 0.437137  (0.345045) | 1.724478  (0.179746) | 0.824397  (0.484938) |
| 24 | Tyrosine | 6.89 (d) | 0.309504 | 0.207572  (0.073353) | 0.19326  (0.05586) | 0.196446  (0.066504) | 0.179877  (0.047129) |
| 25 | Histidine | 7.04 (s) | 0.113046 | 0.096841  (0.031094) | 0.090962  (0.037556) | 0.087591  (0.030612) | 0.082214  (0.032381) |
| 26 | Tyrosine | 7.19 (d) | 0.358837 | 0.229347  (0.088944) | 0.19533  (0.067327) | 0.223322  (0.08532) | 0.181192  (0.053143) |
| 27 | Phenylalanine | 7.33, 7.37, 7.42 (m) | 1.42726 | 1.02554  (0.244563) | 0.85244  (0.267608) | 1.045712  (0.295117) | 0.937641  (0.239304) |
| 28 | Histidine | 7.74 (s) | 0.152447 | 0.082748  (0.028074) | 0.07159  (0.020416) | 0.070099  (0.027363) | 0.069233  (0.025087) |
| 29 | Formate | 8.46 (s) | 0.0523145 | 0.03264  (0.013753) | 0.074833  (0.02983) | 0.032557  (0.011139) | 0.079578  (0.021863) |

**Supplementary Figure S1**. The secretion level of Collagen type III (Col-III) of fibroblasts treated with PBMC secretomes was quantified in culture supernatants using ELISA. Data are expressed as the median values of duplicate samples from independent experiments. Bars show mean ± SD.
